# Correlates of HIV Risk Reduction Self-Efficacy among Youth in South Africa

**DOI:** 10.1100/2012/817315

**Published:** 2012-11-28

**Authors:** Julia Louw, Karl Peltzer, Witness Chirinda

**Affiliations:** ^1^HIV/STI and TB (HAST) Research Programme, Human Sciences Research Council, P.O. Box 339, Bloemfontein 9300, South Africa; ^2^Department of Psychology, University of the Free State, Private Bag X41, Pretoria 0001, South Africa

## Abstract

Even though a decline in HIV prevalence has been reported among South African youth 15–24 from 10.3% in 2005 to 8.6% in 2008, the prevalence remains disproportionately high for females overall in comparison to males. This study examines factors associated by HIV risk reduction self-efficacy of South African youth as part of an evaluation of the impact of loveLife, a youth focused HIV prevention programme. A cross-sectional population-based household survey was conducted with persons of ages 18 to 24 years in four selected provinces in South Africa. Among female respondents (*n* = 1007), factors associated with high self-efficacy in the adjusted model were having a low HIV risk perception, HIV/AIDS stigma, ever using drugs, and having life goals. Male respondents (*n* = 1127) with high self-efficacy were more likely to have been tested for HIV, have concurrent sexual partners, have had a transactional sex partner in lifetime, a low HIV risk perception, difficulty in having condoms, agreed with coercive sex, high relationship control, and had loveLife face-to-face programme participation. The factors identified with high self-efficacy and HIV-sexual risk behaviour may be considered to strengthen youth HIV prevention programmes in South Africa.

## 1. Introduction

South Africa's HIV epidemic remains the largest in the world, with an estimated 5.6 million people living with HIV in 2009 [1]. Young people in particular have the fastest-growing infection rates [2, 3]. They are expected to delay sexual activity and avoid unplanned pregnancy and sexually transmitted infection to safeguard their sexual health [4]. Yet, the complexity of sexual behaviour has been underscored [5] given that HIV risk behaviour is influenced by various factors. Condom use and negotiation of safer sex are reported to be the most effective means of HIV risk reduction [6]. Often, young people struggle to exert control over their emotions when they engage in sexual activity [7], resulting in risky sexual behaviours that lead to HIV infection.

The concept of self-efficacy is defined as having confidence in one's ability to perform a particular behavior, and it has been regarded as an important component of health-related behavioral change [7]. The AIDS risk reduction model in which self-efficacy plays an important role [8] emphasize three stages where a young person has knowledge about a particular safer sex behavior (i.e., using condoms), they then have to think that the behavior is socially acceptable (norms/attitudes) and to believe that they would be able to practice the behavior (self-efficacy) before they actually engage in the behavior [8]. Studies of HIV risk behaviours and sexual health have consistently shown that high self-efficacy for condom use is strongly associated with behaviours of condom use with recent partners and consistent use [6]. A study conducted among young people age 15–24 years reported very high levels of self-efficacy when it came to believing that they could discuss condoms with their partners and refusing sex when they did not want to, but this was not always matched with their actual behaviour [9, 10].

Even though a decline in HIV prevalence has been reported among South African youth 15–24 from 10.3% in 2005 to 8.6% in 2008, the prevalence remains disproportionately high for females overall in comparison to males [2]. The burden of HIV on women, however, varies considerably by region but is heaviest in sub-Saharan Africa with 1.4 times more adult women than men who were living with HIV in 2010 [1]. In South Africa, for example, young females have 3 to 4 times the prevalence of HIV than their male peers [1]. Thus, it is crucial to take into account the gender differences of HIV prevalence [11] and HIV risk behaviours. There is still a dearth of studies that investigate factors that may influence HIV risk reduction self-efficacy by gender. Therefore, this study examines factors associated with HIV risk reduction self-efficacy among South African youth age 15–24 years as part of an evaluation of the impact of loveLife, a youth focused HIV prevention programme.

## 2. Method

### 2.1. Sample and Procedures

A cross-sectional population-based household survey was conducted using a multi-stage stratified cluster sampling approach. In each household all eligible household members were invited to participate and interviewed. The survey included persons of ages 18 to 24 years living in South African households of the four (out of nine) selected provinces, KwaZulu-Natal, Mpumalanga, Eastern Cape and Gauteng Province, providing an urban-rural representation of South Africa. The selection of the provinces was guided by selecting two provinces with the highest HIV prevalence in the country, KwaZulu-Natal and Mpumalanga, and one most urban province (Gauteng) and one rural province (Eastern Cape). Ethical approval for the study was obtained from the HSRC Research Ethics Committee. Participants signed informed consent forms.

### 2.2. Measures

Age, gender, educational and employment status were assessed.

*Contraceptive knowledge* was assessed with 7 items, for example, “Have you heard about the Pill that women can take every day to avoid becoming pregnant?” Response options were “Yes,” or “No.” Cronbach alpha for this contraception knowledge index was 0.70 in this sample.

*HIV knowledge* was assessed with two items: (1) How many people living with HIV do you personally know? And (2) How many people have you personally known (in your lifetime) that have died from AIDS? Responses of the two questions were added up and coded as 0= 0, 1= knows any person living with HIV (PLHIV) or who has died from AIDS, and 2= knows any PLHIV and a person who has died from AIDS. Cronbach alpha for this 2-item HIV knowledge index was 0.73 in this sample.

*“Has goals in life”* was assessed with 6 items such as “I have a plan for the future”; response options were agree or disagree. “Has goals in life” was classified as those who indicated to all 6 items to have any goals. Cronbach alpha for this “goals in life” index was 0.63 in this sample.

*Partner risk reduction self-efficacy* was assessed with 4 items such as “Would you be able to avoid sex any time you did not want it?” Response options were no, probably no, probably yes, yes. Cronbach alpha for this partner risk reduction self-efficacy index was 0.73 in this sample.

*Peer pressure* was assessed with 2 items; (1) “How much pressure you get from your friends to have sexual intercourse, would you say…?” (Response options ranged from 1: “No pressure at all” to 4: “A lot of pressure”) and (2) “I feel pressure from friends to do things I do not want to.” (Response options ranged from 1: “Very often” to 4: “Never”). Low peer pressure was coded 2, medium 3–5, and high 6–8. Cronbach alpha for this peer pressure index was 0.61 in this sample.

*Poverty* was assessed with 6 items on the availability or nonavailability of shelter, fuel or electricity, clean water, medicines or medical treatment, food, and cash income in the past week. Response options ranged from 1: “Not one day” to 4: “Every day of the week” Poverty was defined as higher scores on non-availability of essential items. Cronbach alpha for this poverty index was 0.70 in this sample.

*HIV communication* was assessed with 10 items, specifying 10 different source persons or institutions, for example, “Have you ever talked to your mother or female primary care giver/guardian about HIV/AIDS issues” Responses options were 1: Yes or 2: No. Cronbach alpha for this HIV communication index was 0.75 in this sample. Further, 1 item assessed “Did you talk about using a condom with your latest sexual partner in the last 12 months?” Responses options were 1: Yes or 2: No.

*Accessibility of condoms* was assessed with the question, “How easy is it for you to get condoms if you needed or wanted them?” Response options ranged from 1: very easy to 4: very difficult.

*Relationship control *was assessed with 4 items (for those never in a relationship, they were asked imagine to be) such as “Your partner has more control than you do in important decisions that affect your relationship.” Response options ranged from 1: “Strongly disagree” to 4: “Strongly agree.” Higher scores on lack of relationship power were defined as lack of relationship control. Cronbach alpha for this relationship control index was 0.81 in this sample.

Risk behavior various questions were asked to assess risk behaviour. These included number of lifetime transactional sexual partners, early sexual debut (below 15 years, ever forced to have sex, concurrent sexual relationships, sex with someone who is much older, sexual intercourse frequency, and length of last relationship). 

*Alcohol use* was assessed with the Alcohol Use Disorder Identification Test (AUDIT-C) questionnaire [12], a measure of consumption of alcohol (i.e., the frequency of drinking, the quantity consumed at a typical occasion), and the frequency of heavy episodic drinking (i.e., consumption of five standard drinks, 60 gram alcohol, or more on a single occasion). Because AUDIT is reported to be less sensitive at identifying risk drinking in women [13], the cutoff points of binge drinking for women (4 units) were reduced by one unit as compared with men (5 units), as recommended by Bush et al. [14]. Using a cut off score of 5 or more hazardous or harmful drinking was defined [15, 16]. Cronbach alpha reliability coefficient was in this study for the AUDIT-C 0.91.

*HIV status* was assessed by self-report.

*Prevention programme exposure* was assessed with the following items. Exposure to ever loveLife face to face programmes was assessed with 24 items, for example, Gone to a loveLife clinic, Participated in a loveLife Community Dialogue, or Gone to a loveLife Youth Centre. LoveLife exposure to face-to-face programmes was summed up and coded as 0, 1-2, 3-4, or 5 or more programme exposures. Further, longer-term participation was assessed with having participated in loveLife programmes for at least one year. In addition, loveLife multi-media exposure was assessed with 9 items, for example, “Have you ever watched a loveLife television show?” “Contacted loveLife on facebook” “Heard a loveLife advert on radio.” “Read UNCUT (loveLife) youth magazine.” Response options were 1: Yes or 2: No. The 9 multi-media programmes were summed up and coded as 1: 0-1 media exposures, 2: 2–4, and 3: 5–9 media exposures.

### 2.3. Data Analysis

Both descriptive and inferential statistics were applied in data analysis. Sample weights were calculated after editing the data, and STATA software was used for the analysis. STATA software (svy methods) was used to obtain the estimates of key indicators, significance values (*P* values), and confidence intervals (95% CI) that take into account the complex design and individual sample weights. Computed estimates and odds ratios are reported with 95% confidence intervals and a two-side *P* value of 0.05 used as the cut-off point for statistical significance. Data were checked for normality distribution and outliers. For nonnormal distribution nonparametric tests were used. The Chi-square test was used for comparing nominal variables. Logistic regression analysis was used to study the association between key outcomes (high self-efficacy) and predictor variables. All variables statistically significant at the *P* < .05 level in bivariate analyses were included in the multivariable models.

## 3. Results

### 3.1. Survey Response Rate

A total of 5 768 households were sampled and approached for the interview. Only 94.8% households were valid and among the valid households 93.6% agreed to be interviewed. Only households that indicated that they had a person aged 18 to 24 years were eligible for an individual questionnaire administration. Of the eligible and valid households 47.2% were eligible for an individual interview, 1.3% refused the individual interview, and 2.3% individuals were absent from the household so that the individual interview response rate was 96.4%.

### 3.2. Sample Characteristics

The total samples of young people in the study were 3123, aged 18–24, from four of nine provinces (Eastern Cape, Gauteng, KwaZulu-Natal, and Mpumalanga) in South Africa. From the total sample 1127 and 1007, men and women, respectively, reported to have ever had sex. Overall, among those who had been tested for HIV and indicated their test result 9.3% of the women and 6.2% of the men were HIV positive. Youth programme exposure was assessed in terms of face-to-face participation and media programme exposure, 32.6% had participated in one or more loveLife face-to-face programmes, while more than 80% had been exposed to 2 or more loveLife HIV prevention media programmes (see Table 1).

### 3.3. Self-Efficacy and Condom Use Consistency in South African Females and Males

Among males who indicated that they were not able to avoid sex any time they did not want it, 56.5% use condoms consistently. Males who indicated that they are able to avoid sex any time they did not want it, 58.9% use condoms consistently. These findings were statistical significant. For women though, no statistical significance was reported when asked “would you be able to avoid sex any time you did not want it.” When asked “would you be able to talk about using condoms with your partner?”, of those women who answered no, only 26% are using condoms consistently (versus 41.8% of males) (see Table 2). Generally, across the self-efficacy scale items, more males than females were more likely to use condoms consistently.

### 3.4. Predictors of Self-Efficacy

Univariate analysis found that for males, having been tested for HIV, concurrent sexual partnerships, transactional sex partner, low HIV risk perception, Talked to partner about condom use in the past 12 months, difficulty in getting condoms, acceptable to have coerced sex, high relational control, current tobacco, ever drugs, and participating in few face-face loveLife programmes (1-2) to be associated with high self-esteem.

In the case of females, ever forced to have sex, concurrent sexual partnerships, transactional sex partner in lifetime, low HIV risk perception, talked to partner about condom use in the past 12 months, high peer pressure, stigma, ever drugs and having life goals were associated with high self-efficacy in univariate analysis. 

Multivariate analysis found the following variables to be significantly associated with high self-efficacy for males: having been tested for HIV, concurrent sexual partners, transactional sex partners in life time, low HIV risk perception, difficulty of getting condoms, acceptable to have coerced sex, high relationship control, and participating in 1-2 loveLife face-face programmes. With regards to females, low HIV risk perception, HIV/AIDS stigma, ever drugs and having life goals were associated with high self-efficacy in multivariate analysis (see Table 3).

## 4. Discussion

The findings indicate that, for males, high self-efficacy is significantly associated with knowing your HIV status but only half of the men reported ever testing for HIV. In the case of females, the present study found a significant association between high self-efficacy and having life goals. This finding is similar with a study done among South African youth 15 to 24 years that also found high self-efficacy to be associated with having life goals but in this study it was reported for both young men and women [6]. 

A significant association was also found for young males finding it acceptable to have coerced sex. This probably reflects the higher percentage reported by females than their male counterparts (7.3% versus 1.1%) ever being forced to have sex against their will. This reflects some sense of disempowerment of young females in preventing themselves against HIV infection [17]. As a result it perpetuates social and cultural forces that shape young people's sexual behaviour [18]. Indeed, Varga [19] found that young men seemed to have more freedom to be involved with more than one sexual partner at a time while young women are expected to be faithful to their (one) partner. Thus Varga in her study found that a young man involved with multiple sexual partners was regarded as “*isoka*” (the act of “*ubusoka*”) to indicate the young man's popularity with women something that seemed to be desirable while a young woman with more than one sexual partner was perceived as lacking self-respect [19]. Jewkes et al. [17] would have argued that HIV prevention programmes which emphasize male condoms and male circumcision may further perpetrate young men's freedom in sexual decision making, thus overlooking the vulnerability of young women. This brings into question the effectiveness of existing risk reduction programmes that specifically focus on risk reduction through behavioural interventions for adolescents. Ross [20] paints a rather bleak picture of the efficacy of behavioural intervention. In an update of a recent systematic review of HIV prevention interventions focusing on trials that have included HIV as an outcome, Ross [20] noted that the behavioural interventions conducted through trials were disappointing in terms of the impact on HIV even though some trials showed an impact on other sexual and reproductive health outcomes. The interventions tested were inherently ineffective partly because they were inadequately implemented, or there were problems with the measurement of effectiveness. Perhaps, particular attention needs to be given to the social and sexual norms of the general population among whom the particular target group in this case, young people, live and interact [20].

The present study found that, for males, there was a significant association between high self-efficacy and having concurrent sexual partners as well as having had a transactional sex partner in lifetime. Of grave concern is that high self-efficacy was also significantly associated with those males having difficulty in getting condoms. This is an indication that, even though males reported higher consistent condom use than females, many young men still engage in unprotected sex thus putting themselves and their partners at risk. Varga [21] noted that a predominant theme has been the powerful positive symbolism attached to unprotected sex and negative connotations of condom use. Many women feel stigmatized when insisting on using a condom during sexual intercourse as this is a sign of distrust in their partner [6] or an indication that they are HIV positive. A review done by Marston and King [18] on identifying key themes from various qualitative findings highlighted one theme that specifically relates to this aspect; “condoms are stigmatizing and associated with lack of trust” [18].

Despite an increase in awareness of HIV status in the general population between 15 and 49 year old doubling from 11.9% in 2005 to 24.7% in 2008 [11], unprotected sex with intimate personal partners is nearly always chosen over condom use [21]. A partial explanation for the sexual dynamics perhaps lies in a sociocultural context which has not fostered sexual negotiation skills. A renewed focus on programmes related to self-efficacy programmes in young people is urgently needed. Given the significant finding that amongst males, high self-efficacy is associated with exposure to at least 1 to 2 face-to-face loveLife programmes, evidence do exist that similar intervention strategies focusing on self-efficacy has a positive effect.

The results further confirm loveLife's emphasis on normative and social factors as being one of the issues that young people have to contend with in their constructions of sexuality, and this seems to be particularly true of young women [22, 23]. For instance, more young women report having had sex with a much older partner. This is consistent with the findings from findings in the general population survey [11] where young people reported having partners five or more years older than themselves, and there was a substantive increase from 9.6% in 2005 to 14.5% in 2008. This makes them more vulnerable to HIV infection because they find it difficult to negotiate sex with their older partner [24]. If indeed young women's sexual partnerships with older men are solely aimed at economical gain, initiatives aimed at young women's economic emancipation need further support.

## 5. Limitations

One of the limitations of that study was that it was a cross-sectional study. Furthermore, it was only done in four provinces; thus, the results cannot be generalized.

## Figures and Tables

**Table 1:** Individual, social, structural, contraception, and prevention programme exposure of study sample by gender.

	Male (*N* = 1127)	Female (*N* = 1007)
*Sociodemographics*	*N* (%) or *M* (SD)	*N* (%) or *M* (SD)
Poverty index (range 6–24)	8.3 (3.0)	7.9 (2.6)
≤Grade 10	218 (14.7)	171 (18.0)
Grade 11	233 (17.3)	201 (18.5)
Grade 12 or more	670 (68.0)	632 (63.5)
Student	459 (42.3)	361 (44.0)
Employed	217 (23.0)	114 (11.1)
Unemployed	370 (34.8)	440 (45.0)
Knowledge		
Contraceptive knowledge (range 0–7)	4.7 (1.6)	5.1 (1.5)
Knows person living with HIV and/or died from AIDS		
0	393 (40.0)	272 (36.3)
1 = knows PLHIV or died from AIDS	252 (23.5)	215 (18.2)
2 = knows PLHIV and died from AIDS	475 (36.6)	514 (45.5)
Ever HIV test	556 (48.9)	779 (80.4)

*Prior sexual experiences*		
Early sex (<15)	145 (17.8)	65 (6.9)
Ever forced sex	14 (1.1)	58 (7.3)
Sex with much older partner	70 (4.3)	232 (19.3)
Concurrent sexual partners	126 (6.8)	53 (3.1)
Transactional sex partner in lifetime	103 (7.8)	50 (5.0)
Outcome expectancies		
Low HIV risk perception	294 (23.6)	354 (30.2)
Believes that condom use is a sign of not trusting the partner	259 (15.9)	229 (17.5)

*Sociostructural factors*		
Peer pressure		
Low	350 (41.0)	465 (54.6)
Medium	447 (42.3)	398 (37.5)
High	206 (16.6)	121 (7.9)
Talked with partner about condoms in past 12 months	820 (94.1)	769 (90.7)
HIV communication (range 0–10)	5.6 (2.5)	5.6 (2.2)
HIV/AIDS stigma	118 (5.5)	75 (3.8)
Difficulty of getting condoms (range 1–4)	1.22 (0.6)	1.13 (0.5)
Lack of relationship control (range 4–16)	8.5 (2.3)	8.3 (2.5)
Agreed with statement “It is acceptable to have sex with my sex partner even though my partner does not want to.”	60 (4.0)	43 (2.9)
Hazardous or harmful alcohol use	430 (34.0)	160 (16.4)
Ever drug use	162 (25.4)	32 (2.6)
Current tobacco use	363 (25.4)	69 (5.6)
Has goals in life	728 (72.4)	628 (63.6)

*HIV status *		
Diagnosed HIV positive Diagnosed HIV negative	35 (6.7) 493 (93.3)	64 (9.5) 654 (90.5)
HIV risk reduction self-efficacy	462 (52.7)	488 (56.6)

*Programme exposure*		
One year or more loveLife participation	315 (22.4)	250 (21.5)
loveLife face-to-face participation		
0	700 (70.4)	639 (62.6)
2–4	219 (12.2)	193 (22.1)
5–9	100 (8.0)	71 (4.3)
5 or more	98 (9.3)	101 (11.0)
loveLife multimedia programme exposure		
0-1	174 (18.1)	180 (20.7)
2–4	577 (50.8)	458 (48.2)
5–9	340 (31.1)	331 (31.0)

**Table 2:** Self-efficacy scale responses and condom use consistency.

		Men			Women		
		Consistent condom use			Consistent condom use		
Self-efficacy scale items^∗^		No	Yes	*P*	No	Yes	*P*
Would you be able to avoid sex any time you did not want it?	No Yes	43.5 41.1	56.5 58.9	0.000	57.9 54.4	42.1 45.6	0.352
Would you be able to use a condom every time you have sexual intercourse?	No Yes	46.1 40.4	53.9 59.6	0.000	66.8 51.4	33.2 48.6	0.000
Would you be able to refuse to have sex if your partner will not use a condom?	No Yes	47.3 38.4	52.7 61.6	0.000	65.7 49.8	34.3 50.2	0.000
Would you be able to talk about using condoms with your partner?	No Yes	58.2 32.2	41.8 67.8	0.000	74.0 44.0	26.0 56.0	0.000

^∗^Scale items response options were No, Probably No, Probably Yes, Yes; “No” was coded “No, Probably No, Probably Yes.”

**Table 3:** Association between individual, social and structural variables, loveLife exposure, and high self-efficacy.

	Men		Women	
	UOR (95% CI)	AOR (95% CI)	UOR (95% CI)	AOR (95% CI)
*Sociodemographics*				
Education	1.02 (0.84–1.24)	1.07 (0.78–1.46)	1.27 (0.99–1.62)	1.16 (0.82–1.64)
Student	1.00	1.00	1.00	1.00
Employed	2.35 (0.79–6.96)	2.03 (0.62–6.01)	0.75 (0.35–1.58)	0.66 (0.22–1.95)
Unemployed	0.72 (0.49–1.07)	0.67 (0.33–1.37)	1.13 (0.59–2.18)	1.33 (0.60–2.94)
Poverty index	1.04 (0.95–1.13)	1.11 (0.99–1.25)	1.06 (0.86–1.31)	1.15 (0.91–1.45)
Knowledge				
Knows person living with HIV and/or died from AIDS				
0	1.00	—	1.00	—
1 = knows PLHIV or died from AIDS	0.41 (0.12–1.40)		0.97 (0.54–1.77)	
2 = knows PLHIV and died from AIDS	0.72 (0.45–1.15)		0.72 (0.37–1.39)	
Have been tested for HIV ever?	2.38 (1.41–4.02)^∗∗∗^	2.54 (1.38–4.67)^∗∗^	0.88 (0.55–1.40)	—
Contraceptive knowledge	1.11 (0.75–1.65)	—	1.01 (0.90–1.13)	—

*Prior sexual experiences*				
Early sex (<15)	2.72 (0.72–10.2)	—	0.94 (0.36–2.44)	—
Ever forced sex	0.62 (0.10–3.91)	—	0.31 (0.11–0.86)^∗^	0.27 (0.03–2.03)
Concurrent sexual partners	0.23 (0.12–0.47)^∗∗∗^	0.28 (0.12–0.65)^∗∗^	0.30 (0.11–0.84)^∗^	0.49 (0.16–1.51)
Sex with much older partner	0.34 (0.11–1.12)	—	0.72 (0.45–1.17)	—
Transactional sex partner in lifetime	0.10 (0.05–0.20)^∗∗∗^	0.10 (0.03–0.29)^∗∗∗^	0.09 (0.03–0.27)^∗∗∗^	0.70 (0.17–2.83)
Outcome expectancies				
Low HIV risk perception	1.41 (1.13–1.76)^∗∗^	1.45 (1.04–2.02)^∗^	1.85 (1.47–2.34)^∗∗∗^	2.26 (1.25–4.09)^∗∗^
Beliefs condom use is a sign of not trusting the partner	0.79 (0.42–1.46)	—	0.66 (0.36–1.20)	—

*Sociostructural factors*				
Talked to partner about condom use in the past 12 months	2.50 (1.25–5.00)^∗∗^	1.21 (0.46–2.75)	2.93 (1.55–5.53)^∗∗∗^	
HIV communication	1.07 (0.89–1.30)	—	0.97 (0.83–1.14)	—
Peer pressure				
Low	1.00	—	1.00	1.00
Medium	0.35 (0.14–0.87)		0.68 (0.40–1.15)	0.93 (0.51–1.67)
High	0.23 (0.05–1.13)		0.28 (0.14–0.55)^∗∗∗^	0.89 (0.33–2.30)
Difficulty of getting condoms	0.60 (0.37–0.97)^∗^	0.60 (0.38–0.98)^∗^	0.81 (0.63–1.03)	—
Agreed with statement “It is acceptable to have sex with my sex partner even though my partner does not want to.” (Acceptable to have coerced sex)	0.23 (0.08–0.67)^∗∗^	0.26 (0.07–0.98)^∗^	0.76 (0.23–2.49)	—
HIV/AIDS stigma	0.68 (0.25–1.82)	—	0.24 (0.11–0.50)^∗∗∗^	0.20 (0.06–0.64)^∗∗^
High relationship control	0.46 (0.27–0.79)^∗∗^	0.24 (0.10–0.58)^∗∗^	1.10 (0.72–1.69)	—
Hazardous or harmful drinking (AUDIT > 4)	0.70 (0.44–1.14)	—	0.70 (0.21–2.33)	—
Current tobacco use	0.48 (0.27–0.87)^∗^	0.58 (0.26–1.28)	0.60 (0.13–2.64)	—
Ever drugs	0.35 (0.18–0.66)^∗∗∗^	0.45 (0.17–1.31)	0.10 (0.03–0.39)^∗∗∗^	0.12 (0.02–0.74)^∗^

*Goals*				
Has life goals	2.78 (0.98–7.86)	—	1.85 (1.16–2.96)^∗^	2.98 (1.16–7.63)^∗^

*Programme exposure*				
One year or more loveLife participation	0.85 (0.43–1.71)	—	1.15 (0.63–2.08)	—
loveLife face-to-face participation				
0	1.00	1.00	1.00	—
1-2	0.42 (0.22–0.81)^∗∗^	0.38 (0.16–0.89)^∗^	0.48 (0.14–1.72)	
3-4	0.87 (0.30–2.47)	0.50 (0.22–1.13)	0.62 (0.22–1.74)	
5 or more	1.70 (0.47–6.20)	2.11 (0.46–9.59)	0.81 (0.28–2.33)	
loveLife multimedia programme exposure				
0-1	1.00	—	1.00	—
2–4	0.88 (0.40–1.90)		0.63 (0.34–1.17)	
5–9	0.70 (0.37–1.34)		1.33 (0.54–3.25)	

^∗∗∗^ *P* < .001; ^∗∗^ *P* < .01; ^∗^ *P* < .05.
